# Non-invasive monitoring of adrenocortical physiology in a threatened Australian marsupial, the western quoll (*Dasyurus geoffroii*)

**DOI:** 10.1093/conphys/coz069

**Published:** 2019-10-31

**Authors:** Melissa A Jensen, Katherine E Moseby, David C Paton, Kerry V Fanson

**Affiliations:** 1 School of Biological Sciences, University of Adelaide, Adelaide, South Australia 5005, Australia; 2 Centre for Integrative Ecology, Deakin University, Waurn Ponds, Victoria 3216, Australia

**Keywords:** Adrenal, capture, cortisol, faecal, glucocorticoid, physiological stress response, transport, validation

## Abstract

Reintroduction has become an increasingly important conservation tool in Australia, yet the effects of stress on species during reintroduction programs have received little attention. The use of enzyme immunoassays to measure faecal glucocorticoid metabolites (FGM) is a useful non-invasive technique to monitor adrenal activity but requires validation before they can be reliably used. As part of a large reintroduction project, the goals of this study were to 1) monitor FGM in 53 western quolls (*Dasyurus geoffroii*) following capture from the wild and transfer to a holding facility and use this stressor to biologically validate an enzyme immunoassay; 2) determine if biological factors, such as sex, age, weight or source population affect baseline FGM levels; and 3) examine individual variation in the acute adrenal response of quolls to the capture and transfer associated with reintroductions. We successfully validated an assay that targets glucocorticoid metabolites with a 5α-3β,11β-diol structure and found that sex significantly influenced both baseline and peak FGM output in western quolls, whereas age, weight and source population did not. We also observed considerable variation among individuals in the magnitude and duration of their physiological response to capture and transfer. Using the methods described here, FGM analysis may provide further information about the adrenal activity of the western quoll and improve future conservation efforts for this threatened species.

## Teaser

We monitored faecal glucocorticoid metabolites (FGM) in 53 western quolls following capture. Using this stressor, we validated an assay for monitoring FGM in quolls. On average, FGM peak 24 hours after capture. Females exhibit higher baseline and peak FGM levels than males. Individuals vary in their physiological response to capture.

## Introduction

Reintroduction is becoming an increasingly important conservation tool worldwide, yet the stress experienced by wildlife during reintroduction programs and how this may affect reintroduction outcomes has received little attention (Teixeira *et al.*, 2007). Stress can contribute to an animal's well-being and can affect how wildlife respond to environmental change (Killen *et al.*, 2013; Madliger and Love, 2014; Reeder and Kramer, 2005). Therefore, an understanding of the physiological stress response may be valuable for understanding reintroduction outcomes, reducing the stress experienced during translocations, and potentially increasing the success of reintroduction efforts.

Stressors activate a cascade of events in the hypothalamic–pituitary–adrenal axis in vertebrates. The hypothalamus secretes corticotrophin-releasing hormone, which causes the pituitary gland to release adrenocorticotrophic hormone. This, in turn, increases glucocorticoid release from the adrenal cortex. At baseline levels, glucocorticoids play an important role in many everyday physiological processes, such as regulating circadian rhythms, and promoting healthy immune function and reproduction (Boonstra, 2004; Padgett and Glaser, 2003; Sapolsky, 2002). However, prolonged elevation of glucocorticoid levels caused by chronic stress can have detrimental health effects, such as suppressed reproduction, impaired immune function and decreased cognitive function (McEwen 1998; Sheriff *et al.*, 2009). Therefore, monitoring adrenal function can provide insight into the health and well-being of an animal.

Adrenal function and the physiological stress response of wildlife can be monitored by measuring glucocorticoids (Touma and Palme, 2005). Traditionally, glucocorticoids have been monitored via blood sampling (Möstl and Palme, 2002; Touma and Palme, 2005). However, this technique requires capturing, restraining and sampling blood from the animal. This can be invasive, impractical and disruptive, particularly if intending to repeatedly monitor rare or cryptic species, or those settling into a new environment following reintroduction. Handling can also cause rapid changes in circulating glucocorticoid levels, thus affecting results (Mormède *et al.*, 2007; Möstl and Palme, 2002; Sheriff *et al.*, 2011). Furthermore, because glucocorticoids are released in pulses and follow a circadian rhythm, blood sampling only provides a snapshot of circulating glucocorticoid levels at that point in time (Sheriff*, et al.*, 2011; Touma and Palme, 2005).

The development of non-invasive hormone monitoring techniques allows researchers to monitor adrenocortical activity via glucocorticoid metabolites in faeces, eliminating the need to capture and take blood from the animal (Sheriff*, et al.*, 2011; Touma and Palme, 2005). Consequently, samples can be collected more frequently, providing more information about endocrine function. Faecal glucocorticoid metabolites (FGM) also provide a pooled estimate of circulating glucocorticoid concentrations over time, providing a measure of the animal's overall physiological state (Goymann *et al.*, 1999; Keay *et al.*, 2006; Whitten *et al.*, 1998). Faecal glucocorticoids are metabolized by the body before excretion and both the patterns of steroid metabolism and excretion routes can vary substantially between species, even those that are closely related (Bahr *et al.*, 2000; Palme *et al.*, 2005). Therefore, biologically validating an assay before use is essential for each new species in order to ensure that it detects biologically relevant and expected changes in adrenal activity following a stressful event (Buchanan and Goldsmith, 2004; Goymann, 2012; Palme, 2005; Sheriff*, et al.*, 2011; Touma and Palme, 2005). In addition, glucocorticoid levels can be affected by a variety of biological factors, such as age, sex, reproductive status, body condition and social status (Carnegie *et al.*, 2011; Cavigelli, 1999; Ferreira Raminelli *et al.*, 2001; Sapolsky, 1991; Seraphin *et al.*, 2008; Setchell *et al.*, 2008; Touma *et al.*, 2003; Weingrill *et al.*, 2004; Ziegler *et al.*, 1995), which may confound interpretation of glucocorticoid concentrations (Boonstra, 2004). Therefore, to ensure accurate interpretation of FGM, it is important to establish normative glucocorticoid patterns by determining if and how these variables have an impact on FGM concentration.

Numerous mammal reintroductions have been attempted in Australia (Fischer and Lindenmayer, 2000; Morris *et al.*, 2015; Short and Smith, 1994), but very little is known about the basic stress physiology of Australia's marsupial species (Hing *et al.*, 2014; McDonald, 1977), or how they cope physiologically with these reintroduction programs (Dickens *et al.*, 2010; Teixeira*, et al.*, 2007). The western quoll (*Dasyurus geoffroii*), also known as the chuditch, is a medium-sized marsupial (average adult body weight 900–1300 g) that was once found across 70% of mainland Australia, but is now confined to the forests of southwest Western Australia (Morris *et al.*, 2003; Serena and Soderquist, 2008). The species is nocturnal, carnivorous and solitary. They are currently listed as vulnerable under the Australian Environment Protection and Biodiversity Conservation (EPBC) Act 1999 and have been subject to numerous translocation attempts in Western Australia in order to recover the species (Morris*, et al.*, 2003; Morris*, et al.*, 2015).

As part of a reintroduction of western quolls to an area of their former range in the Ikara-Flinders Ranges National Park in South Australia (Moseby *et al.*, 2016), we aimed to establish a basic understanding of adrenal activity in the western quoll using the non-invasive technique of FGM analysis. We monitored FGMs in 53 western quolls that were captured from the wild and transferred to a captive facility, where they were held for about 19 days prior to reintroduction. This provided a unique opportunity to biologically validate an enzyme immunoassay for monitoring adrenocortical activity in western quolls by demonstrating that FGM reflect the expected peaks in adrenal activity following a stressful event, in this case capture from the wild and transfer to a captive facility. We also determined if biological factors such as sex, age, weight and source population affect FGM levels in this threatened species. Finally, we examined individual variation in the acute adrenal response of quolls to the capture and transfer associated with reintroductions.

## Materials and methods

### Study animals and sample collection

During March and April 2014 and April 2015, 53 western quolls (23 M, 30 F), ranging from 1 to 3 years of age, were wild caught from three source populations in south-west Western Australia (Table 1). Quolls were trapped in 21 cm (W) × 56 cm (L) × 21 cm (H) wire cage traps that were covered with hessian and baited with a mixture of rolled oats, canned sardines and peanut butter. Traps were set along vehicle access tracks in the evening and checked at dawn the following day. We used age-related traits such as pouch status in females and tooth wear in both sexes to determine animal ages (B. Johnson, Western Australian Department of Parks and Wildlife, personal communication, 2014) and weight was recorded at the time of capture. All animals were in good health and none of the females were carrying pouch-young, as trapping was conducted outside of the breeding season. Following capture, each quoll was placed in an individual wooden box (27 cm (W) × 43 cm (L) × 17 cm (H) in size) and transported to a captive facility in Perth in an air-conditioned vehicle (travel time, 1–5 hours). The quolls were housed in captivity for an average of 19 days (range, 11–40 days) in individual 2 m (W) × 3 m (L) × 2.4 m (H) semi-outdoor enclosures. Each enclosure contained a sandy substrate, hollow logs, freshly cut foliage and a nest box. Quolls were fed each evening on a varied diet consisting of dead mice and day-old chickens, boiled egg, fish and omnivore pellets. Water was provided *ad libitum*.

**Table 1:** Numbers of male and female western quolls captured from each source population in late summer—autumn in 2014 and 2015

	**Male**		**Female**	
	**2014**	**2015**	**2014**	**2015**
Fitzgerald River National Park	0	5	0	1
Julimar State Forest	0	8	8	12
Tone-Perup Nature Reserve	10	0	9	0
**TOTAL**	**10**	**13**	**17**	**13**

Scats from each animal were collected from the trap on the morning of their capture and every morning from their individual enclosures while they were held in captivity. An average of 16 samples (range, 8–26) was collected from each individual, for a total of 870 samples collected. Samples were stored in plastic zip-lock bags at −18 °C until extraction of steroids.

### Steroid extraction

All quoll faecal samples were analysed at Deakin University, Geelong, Australia. To extract the steroid metabolites, we weighed out 0.25 g (± 0.01 g) of each wet faecal sample into individual polypropylene tubes, added 2.5 mL of 80% ethanol, and mixed overnight using a multivortex (Palme *et al.*, 2013). Samples were then centrifuged for 10 minutes (Hettich Universal 320 R, Tuttlingen, Germany; RCF = 3000) and the supernatant was decanted into 1.5 mL microcentrifuge tubes.

### Enzyme immunoassay

Faecal glucocorticoid metabolites were measured using an enzyme immunoassay designed to target glucocorticoid metabolites. The antibody, biotinylated steroid and standard were obtained from R. Palme (University of Veterinary Medicine, Vienna; Lab code 37e). The antibody for this group-specific assay was raised against 3β,5α-tetrahydrocorticosterone and targets metabolites with a 5α-3β,11β-diol structure (Touma*, et al.*, 2003). Methods used were similar to those previously described (Fanson *et al.*, 2017; Touma*, et al.*, 2003). Briefly, 96-well microtitre plates were incubated with 250 μL of coating buffer containing Protein A for 24 hours, emptied, then incubated overnight with 300 μL Trizma buffer solution rich in bovine serum albumin. Plates were then emptied and immediately frozen. Just before use, plates were thawed and washed three times. They were then loaded with 50 μL of standard, control or diluted sample extract, followed by 100 μL of enzyme label and antibody. Plates were incubated on a plate shaker overnight at 4 °C. Each plate was then washed four times, and 250 μL of a streptavidin solution (Sigma S2438) was immediately added to each well. Plates were incubated at 4 °C for 45 minutes while shaking. Following this, plates were again washed four times, and 250 μL of tetramethylbenzadine solution was added to each well before returning them to the refrigerator to incubate. After 1.5 hours, the reaction was stopped with 50 μL of sulphuric acid (2 M), and the plates were read at 450 nm with a 620 nm reference filter using an optical density plate reader (BMG FLUOstar Omega). Each faecal sample was run in triplicate and when possible all of an individual’s samples were run on a single plate. The assay was biochemically validated by demonstrating parallelism between a serially diluted extract pool and the standard curve. The intra-assay coefficient of variation (CV) was < 15% (n = 15 replicates) and the inter-assay CV was 18% (n = 36 plates). Metabolite concentrations are expressed as ng/g wet faecal weight.

### Statistical analysis

Data were analysed in R (version 3.3.1, R Core Team 2016). FGM concentrations were log-transformed to meet assumptions of normality and heteroscedasticity. Statistical threshold was set at *P* ≤ 0.05.

#### Biological validation

To determine if the assay could successfully detect biologically relevant changes in adrenal activity, we tested for the presence of a significant FGM peak following a known stressful event. Capture and transfer to a captive facility typically cause an acute stress response in animals; therefore, the use of capture and transfer or similarly stressful events are routinely used to biologically validate FGM assays (Dickens*, et al.*, 2010; Grandin, 1997; Sheriff*, et al.*, 2011; Touma and Palme, 2005). We validated our assay by running a general linear mixed model using the R package ‘lme4’ (Bates *et al.*, 2015) to determine whether FGM levels were significantly elevated post-capture. It was not possible to collect pre-capture samples, nor could we be certain that samples collected from traps reflected pre-capture levels of adrenal activity. However, following the acute stress response to being captured, most animals exhibit a decrease in adrenal activity after a few days. Therefore, we ran pairwise comparisons between each of the first four individual days (when the stress response should be the greatest) and the average of the remaining time in captivity, with a Tukey correction to account for multiple comparisons. Sex and year were also included as fixed effects in the model. Individual ID was included as a random effect to account for repeated sampling from each individual.

#### Effect of biological factors on faecal glucocorticoid metabolite levels

Our second aim was to examine how biological factors affect FGM concentrations. There are several factors that can influence adrenal activity or steroid metabolism, including age, sex and body condition, and it is important to account for these factors before making inferences about stress (Touma and Palme, 2005). We ran separate analyses for baseline and peak FGM values (as identified using the iterative method described below) because they represent different biological functions of the adrenal gland and consequently may be affected by biological factors in different ways. We ran a linear mixed model to determine the effect of sex, age, weight and source population on FGM concentrations (either baseline or peak). Individual identity was included as a random factor. In addition, we also controlled for the effect of year to account for year-to-year variation in study individuals and assays. For significant main effects, we ran pairwise comparisons with a Tukey correction to identify significant differences among groups.

#### Individual variation in faecal glucocorticoid metabolites in response to capture and transfer

Our third aim was to examine individual variation in the acute adrenal response to capture and transfer associated with reintroduction. We used an iterative baseline approach to identify baseline and peak values for each individual quoll (Brown *et al.*, 1994; Fanson*, et al.*, 2017; Wielebnowski *et al.*, 2002). The baseline was calculated using an iterative process excluding points greater than the mean + 2 standard deviations (SD), until no points fell above this threshold. The points remaining below this threshold were considered ‘baseline’, while ‘peaks’ were defined as points exceeding this threshold. All scats that were collected and analysed for FGM concentrations were included in the baseline calculation. This calculation was performed using the package ‘hormLong’ (Fanson and Fanson, 2015). As per other marsupial studies (Fanson*, et al.*, 2017; Hogan *et al.*, 2011; Narayan *et al.*, 2014), we considered any peaks within the first 4 days post-capture to be a biologically relevant indication of capture-related stress.

## Results

### Biological validation

**Figure 1:**
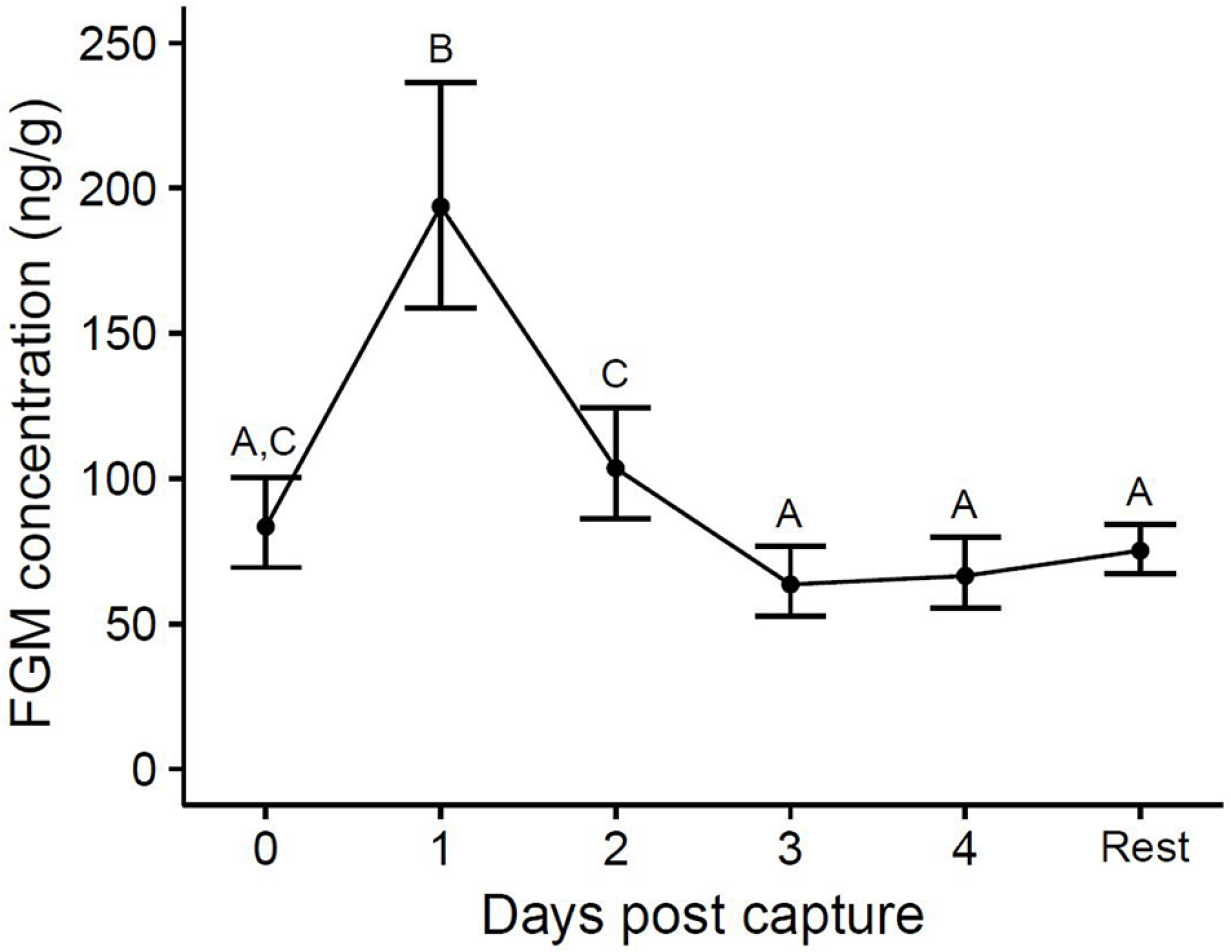
Changes in FGM concentration in western quolls post-capture. Points represent back-transformed least square mean ± 95% CI. ‘Rest’ represents the average of all samples collected after Day 4. Different letters indicate statistically significant differences (*P* < 0.05) among points.

Faecal glucocorticoid metabolite concentrations were significantly elevated in quolls following capture and transfer. The linear mixed model showed that there was a significant effect of time post-capture on FGM concentrations (*F*_5, 818.1_ = 26.41, *P* < 0.001; Fig. 1). FGM were significantly elevated on Days 1 and 2 post-capture, being 3.3- and 1.9-fold higher than the remainder of the time in captivity, respectively.

### Effect of biological factors on faecal glucocorticoid metabolite levels

**Figure 2:**
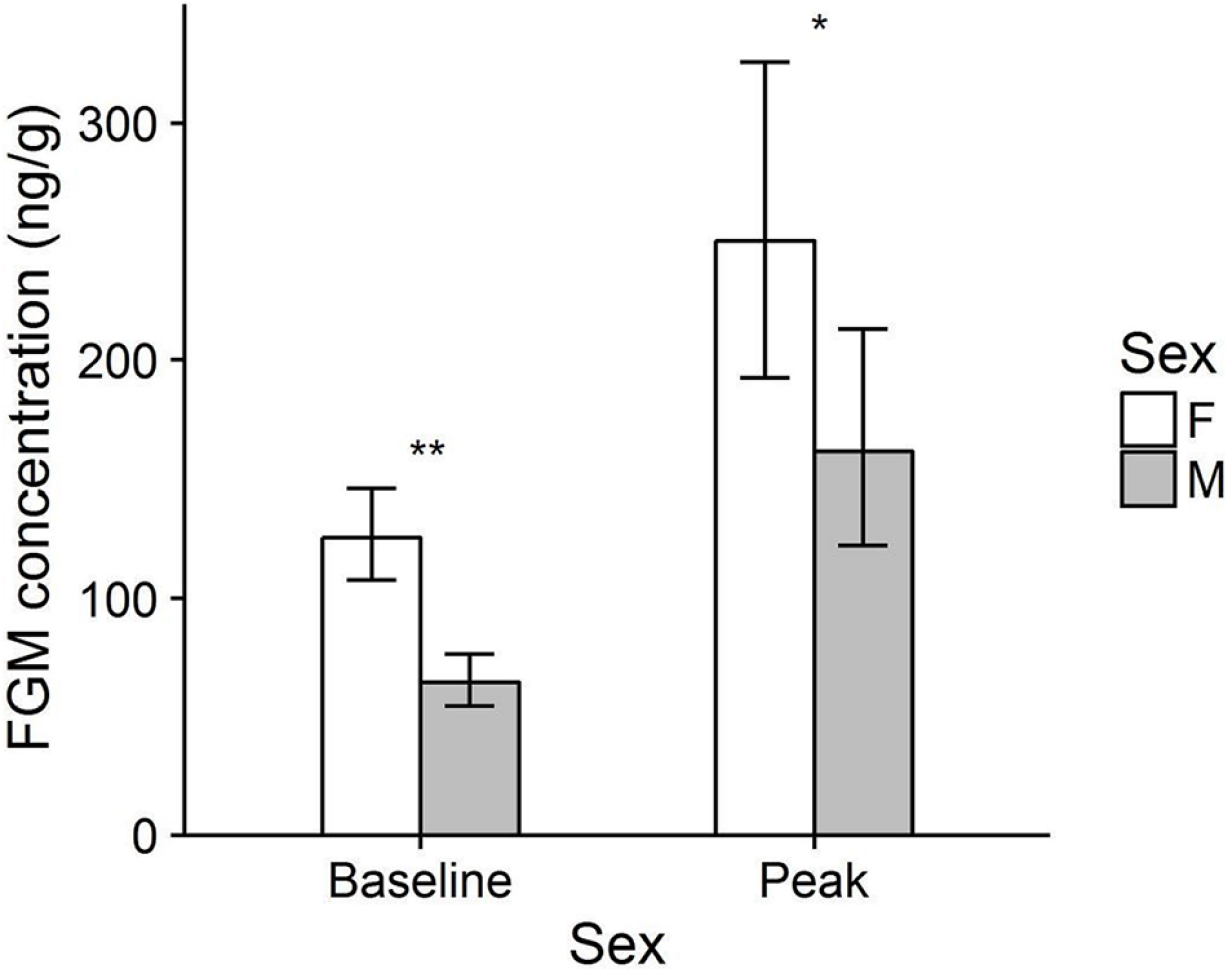
Sex differences in baseline and peak FGM concentrations in western quolls. Bars represent back-transformed least square mean ± 95% CI. ^*^P < 0.05; ^**^P < 0.001.

Of the biological factors considered, only sex had a significant effect on FGM concentrations (Table 2). Female quolls had significantly higher baseline (2-fold higher; t_64.77_ = 3.66, P < 0.001) and peak (1.5-fold higher; *t*_32.22_ = 2.30, *P* = 0.03; Fig. 2) FGM levels than males. Our model also indicated that FGM levels were significantly different between the two study years, with quolls tested in 2015 having higher FGM concentrations than those tested in 2014 (Table 2).

**Table 2:** Results of the linear mixed model showing the effect of biological factors on baseline and peak FGM concentrations in western quolls. Significant values (*P* < 0.05) are indicated in bold

	**Baseline**			** Peak**		
	**DF**	**F**	**P**	**DF**	**F**	**P**
Sex	1, 64.8	13.38	**<0.001**	1, 32.2	5.30	**0.03**
Age	1, 65.8	0.71	0.40	1, 33.3	1.99	0.17
Source population	2, 68.0	0.10	0.91	2, 51.9	0.20	0.82
Weight	1, 66.8	0.01	0.91	1, 44.2	0.75	0.39
Year	1, 67.5	15.64	**<0.001**	1, 45.5	12.83	**<0.001**

### Individual variation in faecal glucocorticoid metabolites in response to capture and transfer

**Figure 3:**
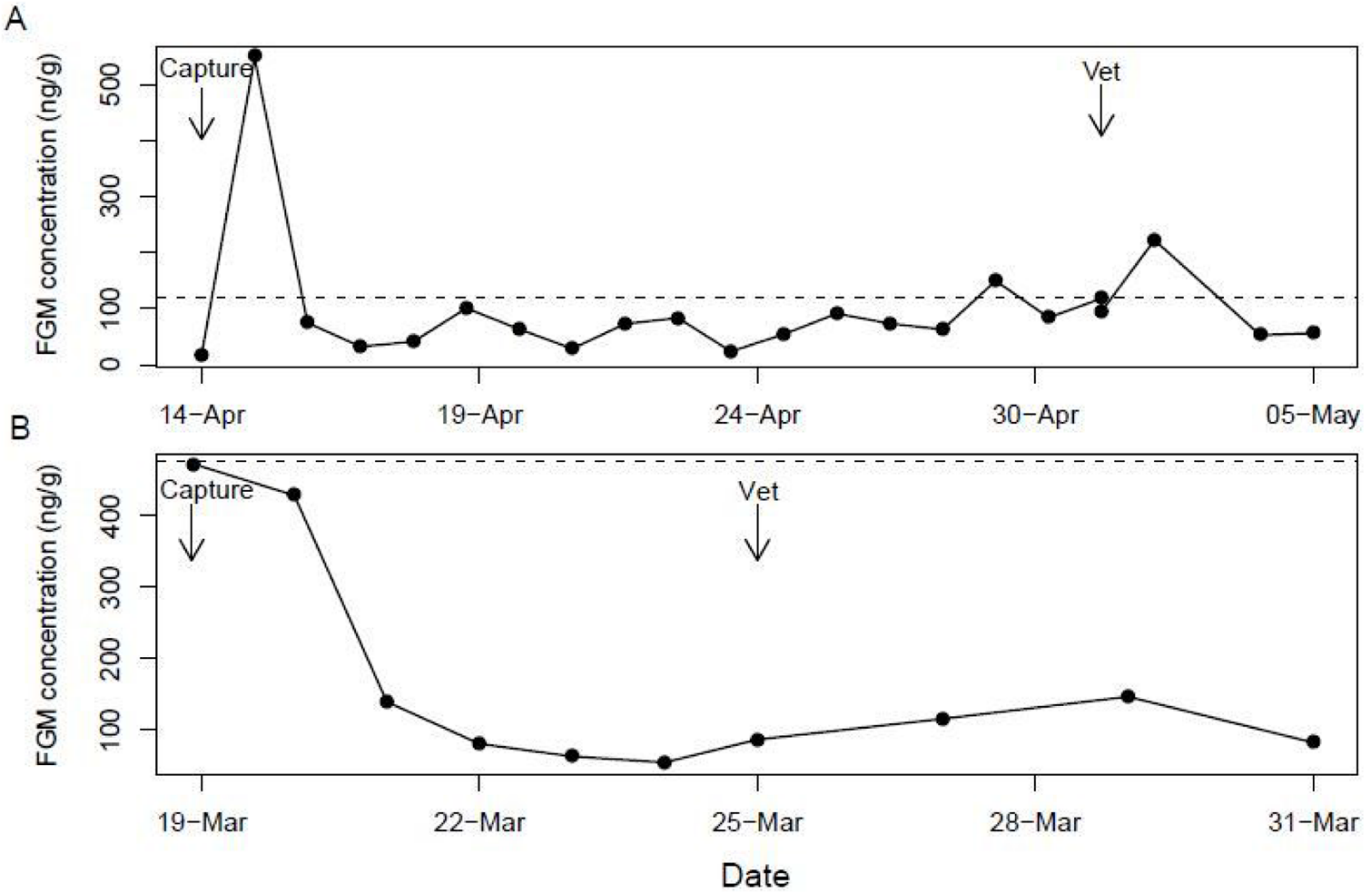
Examples of longitudinal FGM profiles for two quolls following capture from the wild and transfer to a captive facility. Dotted line represents the baseline threshold calculated for that individual via an iterative process excluding all points greater than the mean + 2 SD. Points above this line are considered ‘peaks’. (A) An individual with identified FGM peaks following capture and a vet check. (B) An individual with the expected pattern of FGM following capture, but no points that exceeded the baseline threshold.

Using the iterative baseline approach, we detected FGM peaks in 36 (67.9%) of the 53 quolls within 4 days of capture and transfer (mean ± SD = 1.33 ± 0.89 days; see Fig. 3A for a representative profile). An additional eight quolls (for a total of 83%) showed the expected trend in FGM concentrations, with elevated FGM levels following capture, even though the points did not exceed the 2 SD threshold (see Fig. 3B for a representative profile). These eight cases were all individuals that had fewer samples collected; specifically, fewer baseline samples which results in larger SDs for those individuals. This consequently reduces the sensitivity of the iterative baseline approach to distinguish signal (i.e. peaks) from noise. Post-capture peaks in FGM occurred approximately 24 hours after capture and were on average 3.6-fold (range = 0.69–24.65) higher than an individual’s mean baseline value. For most quolls, the peak was only detected in a single sample and their FGM levels returned to baseline within 24 hours. However, five quolls exhibited peaks in FGM levels that lasted for between 48 and 72 hours, and one quoll exhibited a second peak after 3 days.

## Discussion

Conservation programs such as translocations and reintroductions are important for saving species, but they are also potentially stressful to individuals (Dickens*, et al.*, 2010; Teixeira*, et al.*, 2007). The use of FGM provides a non-invasive tool for monitoring stress physiology during these programs. In this study, we monitored FGMs in 53 western quolls that were captured from the wild and transferred to a captive facility. This provided a unique opportunity to both biologically validate an enzyme immunoassay for monitoring adrenocortical activity in this threatened species and also describe how quolls respond to this component of the reintroduction process.

### Biological validation

Faecal glucocorticoid metabolites were significantly elevated on Days 1 and 2 post-capture compared to the remaining time in captivity, thereby indicating that this assay detects biologically relevant changes in FGM. Unfortunately, we were unable to obtain pre-capture samples for our baseline estimate. Instead, we compared the first 4 days in captivity (acute response to capture) to the remainder of the time in captivity. It is possible that this “captivity baseline” is higher than the pre-capture baseline, so this is a conservative approach for validating the assay. The fact that this assay still detects a post-capture peak suggests it is sensitive enough to detect biologically relevant changes in adrenal activity. This assay targets glucocorticoid metabolites, and has also been shown to work well for monitoring FGM in many other marsupial species, including the eastern grey kangaroo (*Macropus giganteus*), the yellow-bellied glider (*Petaurus australis*), the southern bettong (*Bettongia gaimardi*), as well as another carnivorous marsupial species, the Tasmanian devil (*Sarcophilus harrisii*) (Fanson*, et al.*, 2017).

### Effect of biological factors on faecal glucocorticoid metabolite levels

We examined the effect of age, sex, weight and source population on adrenocortical activity in wild-caught western quolls. We found that only sex had a significant effect, with females having significantly higher baseline and peak FGM levels than males. This pattern has been seen in a number of other Australian marsupial species, including the woylie (*Bettongia penicillata*) (Hing *et al.*, 2017), the southern brown bandicoot (*Isoodon obesulus*) (Dowle *et al.*, 2013), and the greater bilby (*Macrotis lagotis*) (Narayan*, et al.*, 2014), as well as in a range of eutherian mammals including the common marmoset (*Callithrix jacchus*) (Ferreira Raminelli*, et al.*, 2001), the European hare (*Lepus europaeus*) (Teskey-Gerstl *et al.*, 2000), the African wild dog (*Lycaon pictus*) (Creel *et al.*, 1997) and domestic cats and dogs (Schatz and Palme, 2001). This sex difference may either be due to differences in adrenal activity or differences in the metabolism and excretion of glucocorticoids.

We also found a difference in FGM levels between the 2 years of this study, with animals in 2015 having significantly higher FGM concentrations than those in 2014. This may just reflect sampling variation, because different individuals were sampled in different years. It might also be due to differences in biological factors that can affect adrenal activity (e.g. climate, food availability, predation pressure and/or competition) or slightly longer travel times experienced by six individuals in 2015. Alternatively, samples from each year were analysed about a year apart, so this difference could reflect subtle drift due to changes in water pH, new reagents or laboratory temperature (Feswick *et al.*, 2014).

### Individual variation in faecal glucocorticoid metabolites in response to capture and transfer

Following capture, FGM concentrations increased in 44 (83%) of 53 western quolls. For eight of these individuals, the increase was not classified as a peak using the iterative baseline calculation with a threshold criterion of 2 SD, but the pattern of FGM was similar (Fig 3). For most individuals, the peak occurred in a single sample, suggesting an acute response to the capture and transfer procedure. Only five individuals exhibited post-capture peaks in more than one sample. On average, FGM concentrations peaked about 24 hours after capture. The delay between changes in circulating hormone levels and changes in FGM levels depends on the time it takes digesta to travel from the duodenum to the rectum (Palme *et al.*, 1996). The time-lag observed in this study is consistent with other studies of carnivorous species (Keeley *et al.*, 2012; Touma and Palme, 2005; Young *et al.*, 2004). There was considerable variation in the magnitude of the adrenal response to capture and transfer, with the increase in FGM concentrations ranging from 0.69- to 24.65-fold above baseline. Only nine quolls did not exhibit a distinct increase in FGM following capture and transfer.

Methodological limitations likely contributed to some of the observed variation among individuals. First, as mentioned above, sample collection was more difficult for some animals than others. While some quolls had almost daily samples, others had several gaps in sample collection. Not only does this mean that the peak may have occurred in a sample that was not collected, but it also reduces the sensitivity of the iterative baseline method to identify peaks. If there is extensive sampling and thus a robust estimate of an individual's baseline FGM values, then standard error is lower, and peaks are more easily detected. Second, it was not possible to collect samples prior to the quoll's capture from the wild. Therefore, it is not possible to gauge how each individual's FGM concentrations in captivity compare to pre-capture levels.

Nonetheless, there is still considerable variation among individuals in the magnitude and duration of their physiological response to capture. Individuals can vary considerably in both their perception of and physiological response to stressful events (Cockrem, 2013, Sapolsky, 1994). These differences may be due to life experiences or genetic variation (Cockrem, 2013). It is possible that some quolls were not as stressed by the capture and transfer process and coped differently to others.

Two distinct stress coping styles have been recognized across species and have been associated with a suite of behavioural and physiological characteristics which may affect aspects critical to reintroduction success, including survival, dispersal and reproduction (Koolhaas *et al.*, 1999). ‘Proactive’ individuals tend to exhibit lower physiological stress responses and typically respond to stressors with a bold, aggressive fight-or-flight behavioural response, whereas ‘reactive’ individuals respond to stressors with timid, risk adverse and freezing behaviours and exhibit higher physiological stress responses (Koolhaas*, et al.*, 1999). Generally, proactive individuals are more successful in constant or familiar environments, whilst reactive individuals, who are more cautious and less likely to engage in risk-taking behaviours, tend to be more successful in changing or unpredictable environments (Cockrem, 2005), such as those experienced during reintroductions. Therefore, understanding the significance of this individual variation may be important for improving the success of reintroduction efforts.

## Conclusion

We have biologically validated an assay for monitoring adrenal activity in western quolls which can now be used to develop a better understanding of normal versus stress-induced changes in adrenal activity during conservation programs for this species. Importantly, this method does not require blood sampling or restraint of animals beyond routine monitoring events. This study has demonstrated that sex needs to be considered when FGM data are interpreted, as female western quolls exhibit higher baseline and peak FGM levels than males. How sex differences affect the physiological stress response of individuals during reintroduction is understudied (Teixeira*, et al.*, 2007), and would be an interesting avenue for further research. Using FGM analysis as part of conservation management studies may provide further information about the adrenal activity of the western quoll and improve future conservation efforts for this threatened species. Furthermore, recognising and incorporating individual variation in stress responsiveness into reintroduction programs could help improve reintroduction success.
